# Stress Responses in Down Syndrome Neurodegeneration: State of the Art and Therapeutic Molecules

**DOI:** 10.3390/biom11020266

**Published:** 2021-02-11

**Authors:** Chiara Lanzillotta, Fabio Di Domenico

**Affiliations:** Department of Biochemical Sciences “A. Rossi Fanelli”, Laboratory Affiliated to Istituto Pasteur Italia-Fondazione Cenci Bolognetti, Sapienza University of Rome, 00185 Rome, Italy; chiara.lanzillotta@uniroma1.it

**Keywords:** Down syndrome, neurodegeneration, Alzheimer disease, antioxidant response, unfolded protein response, ubiquitin proteasome system, autophagy

## Abstract

Down syndrome (DS) is the most common genomic disorder characterized by the increased incidence of developing early Alzheimer’s disease (AD). In DS, the triplication of genes on chromosome 21 is intimately associated with the increase of AD pathological hallmarks and with the development of brain redox imbalance and aberrant proteostasis. Increasing evidence has recently shown that oxidative stress (OS), associated with mitochondrial dysfunction and with the failure of antioxidant responses (e.g., SOD1 and Nrf2), is an early signature of DS, promoting protein oxidation and the formation of toxic protein aggregates. In turn, systems involved in the surveillance of protein synthesis/folding/degradation mechanisms, such as the integrated stress response (ISR), the unfolded stress response (UPR), and autophagy, are impaired in DS, thus exacerbating brain damage. A number of pre-clinical and clinical studies have been applied to the context of DS with the aim of rescuing redox balance and proteostasis by boosting the antioxidant response and/or inducing the mechanisms of protein re-folding and clearance, and at final of reducing cognitive decline. So far, such therapeutic approaches demonstrated their efficacy in reverting several aspects of DS phenotype in murine models, however, additional studies aimed to translate these approaches in clinical practice are still needed.

## 1. Introduction

Neurodegeneration is defined as the progressive, irreversible loss of neurons, which may affect either the peripheral or central nervous system (CNS). As the neuronal structures worsen, a gradual and progressive loss of cognitive and/or motor skills arises, thus resulting in mental impairment, functional loss, and debilitation [1]. Compelling evidence suggests the existence of common clinical and pathological features between different neurodegenerative diseases (NDDs) among which the most representative are the loss of redox balance and increased oxidative stress, abnormal protein aggregation, proteasomal and/or autophagic dysfunction, inflammation, neuronal apoptosis, and mitochondrial dysfunction [2,3,4,5,6]. Increased oxidative stress, protein aggregation, and the failure of protein degradation pathways are intimately linked factors leading to altered protein homeostasis (proteostasis) [7,8]. Proteostasis is essential for cell health and viability and is ensured by the coordinated regulation of protein translation, folding, trafficking, and degradation. When the equilibrium among these mechanisms is lost, aberrant proteostasis occurs and this represents a central molecular hallmark of aging and NDDs [2,9,10]. Neuronal cells hold a broad array of responses to cope with stress conditions including endogenous antioxidant responses (AOR), protein quality control (PQC) systems, and protein degradation pathways [11,12,13,14,15]. The above-mentioned defensive mechanisms are linked to each other and share common molecular processes; therefore, their induction, as well as their dysfunction is frequently reciprocal. Antioxidant responses represent a powerful protective mechanism against the accumulation of pro-oxidant species, thus helping to maintain the redox balance in the cell [1,7]. Endogenous antioxidant responses include a number of tools, such as superoxide dismutase, the peroxidases, the glutathione redox cycle, or the nuclear factor erythroid 2-related factor 2 (Nrf2)-related response, involved in the detoxification of pro-oxidant species [3]. However, if the amount of reactive oxygen species (ROS) overwhelms the antioxidant capacity of the cell, oxidative stress occurs. A key contributor of NDDs is the redox imbalance of neurons due to increased production of free radicals and damaging species, and/or the malfunction of antioxidant defense [16]. Traditionally, pro-oxidant environments have generally been considered to promote the functional impairment of cells and tissues by damaging structural and functional biomolecules. One of the major consequences of oxidative stress is the oxidation of proteins and the formation of large protein aggregates, which are often toxic to cells if allowed to accumulate [17]. The presence of insoluble aggregates is one of the principal characteristics of NDDs, as in the case of Alzheimer’s disease (AD), Down syndrome (DS), Parkinson’s disease (PD), and amyotrophic lateral sclerosis (ALS) [7,17]. Neuronal PQC systems serves to detect and remove structurally altered proteins, to deal with misfolded/damaged proteins and to prevent their detrimental aggregation. The neuronal PQC systems involve the unfolded protein response (UPR), which is induced if the proteostasis in the endoplasmic reticulum (ER) is disturbed; the heat shock response, which leads to the accumulation of a conserved set of chaperon proteins, and the integrated stress response (ISR), which serves as a common downstream response for challenges ranging from ER stress to amino acid deprivation and viral infection [11,12,18]. In acute transient stress conditions, the adaptive effects of the PQC systems balance proteostasis due to the regulation of protein folding/synthesis and to the induction of cell survival mechanisms. In contrast, during persistent chronic stress conditions, a prolonged activation of PQC systems occurs leading to the sustained reduction of protein synthesis and to the activation of cell death pathways. Recently, a role for the aberrant induction of protein PQC systems was postulated in different NDDs [13]. When PQC pathways fail misfolded proteins are targeted for degradation via ER associated degradation (ERAD) in the cytosol [7,19]. The two principal ways of intracellular protein degradation belonging to ERAD are the ubiquitin-proteasome system (UPS) and autophagy. UPS and autophagy are important mechanisms for the degradation of abnormal, misfolded, and aggregated proteins, and for the recycle of resulting macromolecules. These pathways play an essential role in tissue remodeling, cell survival, and regeneration, while their inactivation may lead to extensive cell death due to the lack of clearance of toxic aggregates in the cytoplasm [15,20,21,22,23,24]. Neurons are vulnerable to the impairment of protein degradation pathways and a progressive decline of both proteasome activity and autophagic induction has been observed in several NDDs, thus resulting in proteotoxicity, chronic ER stress, and ultimately to neuronal loss [6,11,25].

The overall objective of this review is to examine the state of the art of stress response pathways in DS, a peculiar member of the family of NDDs. Further, we will discuss new curative approaches to this neurodegenerative disorder, related to novel therapeutic molecules aimed to reduce OS, potentiate protein surveillance and re-folding mechanisms, and boost protein degradative pathways, which together rescue the cell redox balance and proteostasis, and as well as cognitive decline.

## 2. Brain Pathology in Down Syndrome

DS is the most common genomic disorder caused by the trisomy of chromosome 21 (hsa21) affecting about 6 million people worldwide [26,27]. The incidence of age-related cognitive decline and dementia is higher in individuals with DS than in the general population, and progressive cognitive impairment develops at a far earlier age: the median age of dementia onset across all reported studies is below 60 years [27,28,29]. DS individuals are more prone to developing certain health conditions among which hypothyroidism, autoimmune diseases, epilepsy, hematological disorders, recurrent infections, anxiety disorders, and early onset of AD [29]. Recent advances in health care and the management of co-occurring illnesses have increased the life expectancy of people with DS [30,31] to 55-60 years of age [28,32,33]. To explain the biological perturbations underlying the phenotypic manifestations of DS, two main hypotheses have been proposed: first, a specific gene dosage effect, which includes both the direct consequences of overexpressed HSA21 genes and the downstream effects of such overexpression; and second, developmental instability, by which the unspecific alteration of gene expression from the extra HSA21 results in the disruption of overall biological homeostasis [34,35]. Thus, DS can be defined as a multifactorial disease where an abnormal expression of trisomic genes arises not only from genetic but also from epigenomic factors.

The simplest effect of trisomy is the direct effect of an increased dosage of a single HSA21 gene. The additional copy of the gene that encodes amyloid precursor protein (APP), increases susceptibility to early onset AD in individuals with DS by increasing the levels of amyloid-β (Aβ), that misfolds and accumulates in the brain of people with DS and AD [36,37]. Aβ PET studies in DS have identified a distinct pattern of amyloid deposition, which begin predictably in mid-life [38,39] as confirmed by autopsy studies of DS [40,41]. However, DS brains exhibit Aβ plaques since 12–13 years of age, mainly in the form of diffuse plaques, which are also observed at pre-clinical and prodromal stages of sporadic AD [36,37,42,43]. In DS subjects, aged > 40 years, levels of cortical Aβ deposition are similar to those seen in late onset AD and demonstrate cored neuritic plaques, which have high significance for neuropathological diagnostic purposes [29,36,44,45,46]. Remarkably, autoptic DS brain display the presence of isomerized, racemized, truncated, pyroglutamate, and oxidized Aβ, indicating its accumulation of different post-translational modified forms [47]. Moreover, trisomy of chromosome 21 results in increased gene dosage to many other genes beyond APP that may play a critical role in DS neuropathology. Among triplicated genes, both dual specificity tyrosine phosphorylation regulated kinase 1A (DYRK1A) and the regulator of calcineurin 1 (RCAN1) have a well-established role in the aberrant phosphorylation of tau protein, which is one of the main mechanisms underlying the formation of toxic neurofibrillary tangles (NFTs) in AD [48,49]. Studies on autoptic brain samples have shown that progression of NFTs in DS adults follows a similar staging as in AD, starting in the cortex region, and then spreading to hippocampus, inferior temporal cortex and neocortex [50]. The accumulation of Aβ, NFT and dysfunctional/damaged protein aggregate in DS, is strongly associated with the increase of OS and the aberrant regulation of the proteostasis network as described in the latter chapters of this review.

In the last few years, brain hypoglycemia and alterations of the brain insulin signaling pathway, including brain insulin resistance, are emerging as common mechanisms of neurodegeneration in DS and AD [51,52,53]. DS individuals are four times more likely to develop diabetes than the general population [54,55] and the onset of diabetes occurs earlier in children with DS than in the other children [54]. Recently, we reported the accumulation of markers of brain insulin resistance, such as reduced IR protein levels and increased IRS1 inhibition, in the frontal cortex of DS subject (less than 40 years of age) prior the development of AD [51], suggesting that brain insulin resistance could occur early in DS and persist with age. Due to the important role of insulin signaling pathway in the regulation of brain functions, the development of brain insulin resistance seems to be highly implicated in the promotion of AD-like dementia in DS, representing a key pathological event. Moreover, recent evidence from our lab suggest a role for the nutrient-related dynamic changes of O-GlcNAcylation in the progression of DS neuropathology [56].

## 3. Stress Responses in Down Syndrome Brain

### 3.1. DS Humans and Murine Models Employed in the Study of Stress Response Pathways

The investigation of the role of stress response mechanisms and of the proteostasis network in DS neuropathology has attracted great attention in the scientific community in the last year. To this regard, the analysis of autoptic brain samples from DS subjects of different ages allowed to define the profiling of alterations underlying the neurodegenerative process in DS since early stages [29,57,58]. In addition, the analysis of cells, not belonging to CNS (e.g., fibroblasts or peripheral blood mononuclear cells) from DS living individuals, allowed researchers to deeply investigate the molecular mechanisms linking trisomy 21 and aberrant proteostasis and to presume their potential involvement in brain pathology [59,60]. Intriguingly, a number of different DS murine models have been also employed in the study of the redox and protein homeostasis pathways of DS brain [61,62,63,64]. In particular, the majority of the studies involving mice took advantage of the Ts65Dn model, which carries a freely segregating supernumerary chromosome composed of the majority of the Mmu16 syntenic segment translocated to the pericentromeric region of mouse chromosome 17 (Mmu17) [65]. Ts65Dn animals are trisomic for 122 Hsa21 orthologue genes found on Mmu16 between *App* and *Zbtb21*, but also for 43 protein-coding genes located on Mmu17 that are not found on Hsa21 [66]. The Ts65Dn model has been largely used to investigate AD-like pathology in DS, as well as the Ts2Cje, which has a similar gene content and resulted from a Robertsonian translocation event in the Ts65Dn between Mmu12 and the supernumerary chromosome [67]. The Ts1Cje mouse model was generated by the translocation of a region of Mmu16 between *Sod1* and *Mx1* with the very distal region of Mmu12. The Tc1 mouse model carries a freely segregating supernumerary copy of Hsa21 in addition to the normal complement of mouse chromosomes [68]. Around 175 Hsa21 protein-coding genes are functionally trisomic but the APP gene on the Tc1 trans-chromosome was disrupted and no full-length human APP transcript or protein is produced [69]. Recently, the Dp(16)1Yey murine model has been generated and this carries a duplication of the entire Mmu16 syntenic segment [70] with its trisomic region that consists of the entire Hsa21- relevant complement of Mmu16 genes.

### 3.2. Oxidative Stress and Antioxidant Responses

An increasing number of studies have recently shown that increased OS is an early pathological characteristic of the DS brain and is involved in the onset and progression of AD due to the deregulation of gene/protein expression associated with HSA21 [71,72,73,74]. OS indicates a condition where pro-oxidant species overwhelm the cellular antioxidant defense system, by an increase of ROS production and/or by a decrease in the antioxidant response [17]. Superoxide anion (O_2_^•−^), hydrogen peroxide (H_2_O_2_), and hydroxyl radical (HO^•^) are constantly produced as by-products of aerobic respiration and numerous other catabolic/anabolic processes. The CNS contains high levels of fatty acids, which, in the presence of high metabolic flux, represent fertile soil for the initiation of lipid peroxidation reactions. These, in turn, are responsible for generating increasing amounts of free radicals, as well as, highly reactive products, such as 4-hydroxynonenal (HNE) [75]. As consequence, neuronal cells are greatly susceptible to redox imbalance and the accumulation of oxidative damage [16]. The “oxidative stress theory” of aging, by Harman in 1956, purports that a progressive and irremediable accumulation of oxidative damage influences on critical aspects of senescence, contributing to impaired physiological function, increased incidence of diseases, along with the reduction in life span [76]. Therefore, the pro-oxidant state observed at early ages in DS could be associated with the accelerated aging phenotype and with the development of cognitive impairment. Studies from our group and others demonstrated that redox imbalance is a primary event in DS phenotype [77,78,79,80,81,82,83]. In particular, increased OS levels reported in the fetal DS brain may negatively affect development [84]. Furthermore in DS there is extensively literature describing the accumulation of 8-hydroxy-2’-deoxyguanosine (8-OHdG) oxidized protein, an increase of 3-nitrotyrosine in the cytoplasm of cerebral neurons in DS [85], an increase of protein carbonyls in peripheral blood mononucleate cells from children with DS [59], and the escalation of protein oxidation in the amniotic fluid from mothers carrying a DS fetus [82]. The alteration of redox balance was also observed early in peripheral samples from DS individuals, reinforcing the hypothesis of a pro-oxidant state occurring early in DS [86,87]. In later stages of DS, OS contributes to the neurodegenerative phenomena [88,89]. Interestingly, data derived from DS frontal cortex reported increased total levels of protein carbonyls (PC), prior the development of AD (<40 years old), and of protein bound HNE prior and after the development of AD [8,79]. Redox proteomics analysis revealed that a number of proteins were identified to be oxidatively modified in DS brain involved in intracellular processes such as (i) neuronal trafficking; (ii) the proteostasis network; (iii) energy metabolism; and (iv) mitochondrial function [8,79]. Intriguingly, these processes are associated with adenosine triphosphate (ATP) consumption, and mitochondrial impairment is one of the earliest events in neurodegeneration, which promotes an increase of ROS. Mitochondrial dysfunction has been reported in pre-clinical models of DS and in primary cell cultures from DS individuals [49,81,90,91]. In particular, mitochondrial ROS overproduction has been identified in trisomic human skin fibroblasts, where it was linked to a deficiency of mitochondrial complex I, ATP synthase, ADP/ATP translocators, and adenylate kinase activities [92,93]. Alterations of mitochondrial DNA and of its repair systems were also reported in in DS brain tissue and in fibroblasts from DS individuals [94,95].

**Figure 1:**
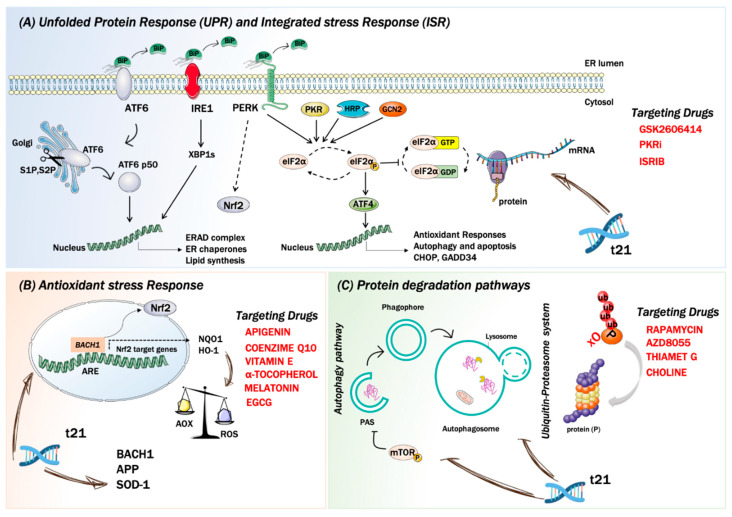
Stress responses in down syndrome neuropathology and therapeutic approaches. Panel (**A**) The unfolded stress response (UPR) and the integrated stress response (ISR) are dysregulated in Down syndrome (DS) and contribute to neurodegenerative processes. Under homeostatic conditions, IRE1, PERK, and ATF6 (UPR sensors) are anchored to the ER membrane by association with BiP. Upon activation of the UPR, BiP releases the effectors of the three branches of the UPR. (**A**) In the pro-survival, adaptive response, IRE1 dimerizes and autophosphorylates. Phosphorylated IRE1 activates XBP1, which in turn is translocated into the nucleus to upregulate the transcription of other adaptive UPR genes. Chronic activation of the UPR leads to pro-apoptotic signals. IRE1 induces apoptosis by activating ASK1 and consequently JNK. ATF6, translocate into the nucleus to enhance transcription of UPR genes. PERK is the only kinase of the UPR that overlap with ISR sensor kinases. PERK dimerizes, autophosphorylates, and targets Nrf2 and eIF2α. PKR, GCN2, and HRI (ISR sensors) once activated converge on the eIF2a phosphorylation, to elicit a translational and transcriptional stress response. Thus, inhibiting the initiation of translation, shutting down protein synthesis, and therefore reducing the load of proteins at the ER. Phosphorylation of eIF2α also triggers the translation of specific mRNAs, including key transcription factors, such as ATF4. In red are reported three targeting drugs used in Down syndrome (DS) acting at three different levels: GSK2606414 on PERK, ISRIB on eIF2a and PKRi on PKR. Panel (**B**) Increased OS is an early pathological characteristic of DS brain and is involved in the onset and progression of AD. In DS a number of genes, directly or indirectly, promote free radical production and alter redox homeostasis. Among these, SOD1, APP, and BACH1 has been recognized as ROS inducers. In DS Bach1 competes, in the nucleus, with Nrf2 for the binding to the antioxidant response elements (ARE) resulting in the increase of OS. In red are reported different antioxidant compounds used in DS both in pre-clinical and clinical study: Apigenin, Coenzyme Q10, Vitamin E, α tocopherol, Melatonin and EGCG. Panel (**C**) The UPS and autophagy are two main pathways involved in protein degradation and are emerged as a prominent disrupted mechanism involved in DS neuropathology. On the left a schematic representation of the autophagosome nucleation/maturation process is shown. Under pathological conditions, the hyperactivation of mTOR mediated by the phosphorylation on Ser2448 is responsible for autophagy inhibition. On the right a schematic representation of the ubiquitin proteasome system is shown. Most of the proteins targeted for proteasomal degradation are covalently modified with ubiquitin. In red are reported three compounds used in DS to rescue autophagy: Rapamycin, Choline and AZD8055.

Noteworthy, the mechanisms responsible for the increase of OS levels in DS may be searched by mapping HSA21, where a number of genes, directly or indirectly, promote free radical production and alter the redox homeostasis of brain cells (Figure 1). Among these, SOD1, APP, carbonyl reductase, and the transcription factor BTB and CNC homology 1 (Bach1) have been recognized as ROS inducers [73,74]. SOD1 catalyzes the dismutation of O_2_^•−^ to O_2_ and H_2_O_2_, the latter in turn is neutralized by catalase (CAT) and by glutathione peroxidase (GPX) to water [96]. Since CAT and GPX are expressed at lower levels in brain compared with other tissues, this reduced ‘buffer’ activity may contribute to the ineffective removal of increasing levels of H_2_O_2_ in DS [89]. In turn, accumulation of H_2_O_2_, in the presence of Fe(II) or Cu(I), leads to hydroxyl radical formation that damage membrane lipids, proteins, and nucleic acids [17]. SOD1 protein levels were increased about 1.5-fold in DS brain and peripheral tissues [59,79]. Further, SOD1 was shown to be targeted by oxidative damage favoring its increased aggregation [79]. However, increased OS in fetal the DS brain could not only be a consequence of SOD1 overexpression, which alone might not explain the generalized increase in oxidative damage [97]. Thus, additional triplicated genes may be involved in the increased susceptibility of DS cells to the accumulation of oxidative damage. APP overexpression leads to the increased production of Aβ peptide and both full-length APP and Aβ are neurotoxic and may promote increased ROS production by interfering with mitochondrial functionality [28,29,36,77,88,98]. Carbonyl reductase catalyzes the reduction of free carbonyl compounds to their corresponding alcohols. Protein carbonyls, including reactive aldehydes such as HNE, can also be detoxified by aldehyde dehydrogenase, which catalyzes their oxidation to carboxylic acids [72]. Protein levels of both these enzymes were found to be increased in different brain regions of both DS and AD cases, indexing the cell response to increase carbonyl production [99]. *BACH1* gene encode for a basic leucine zipper protein belonging to the cap’n’collar family that function as a transcription repressor [74]. In particular, Bach1 competes, in the nucleus, with Nrf2 for the binding to the antioxidant response elements (ARE). Nrf2, through activation of ARE of DNA, mediates induction of multiple antioxidant enzymes such as NADPH quinone oxidoreductase 1 (NQO1), heme oxygenase 1 (HO-1) and numerous constituents of the glutathione pathway [3,100]. Under normal conditions, Keap1, a cysteine-rich protein that senses redox changes in the cell, binds to Nrf2 leading to its retention in the cytosol and causing its proteasomal degradation [61]. Under OS, conformational changes in Keap1 lead to its dissociation from the Nrf2-Keap1 complex and to the translocation of free Nrf2 into the nucleus, where it binds to ARE regions in the genome, to activate the expression of stress response genes [3]. The release of Nrf2 from Keap1 is also achieved by the phosphorylation of Nrf2 on Ser40. Among kinases, PKCs (iota, delta) casein kinase-2 (CK2), phosphatidylinositide-3-kinases (PI3K) c-Jun N-terminal kinase (JNK), and extracellular regulated kinase (ERK) have been reported to be involved in Nrf2 phosphorylation [3]. In addition, the phosphorylation of Nrf2 on Ser40 is also achieved under the activation of the protein kinase R-like ER kinase (PERK), thus strengthening the link between ER stress responses and antioxidant responses [13,61] (Figure 1). Nuclear Bach1, by binding to AREs, displaces Nrf2 and act primarily as a transcriptional repressor for antioxidant genes such as HO-1 and NQO1 [73,74]. Recent data from our laboratory demonstrated the increase of Bach1 in the brain of DS cases and DS with AD [61,73]. The overexpression of Bach1 was associated with decreased Nrf2 expression levels, reduced phosphorylation at Ser40 associated with a reduction in the induction of antioxidant genes and thus increased OS levels [61]. The depletion of Nrf2 antioxidant response ensues in DS brain and contribute to neuropathology, as observed in AD. However, the reduction of Nrf2 active form is observed very early, as an effect of Bach1 triplication. Similar data, concerning the ratio of Nrf2/Bach1 was obtained in DS mice and in PBMCs derived from children with DS [59,61,73]. On the contrary, Zamponi and collaborators reported Nrf2 activation in human astrocytes and fibroblast from DS [101]. Such a discrepancy could be due to differential tissue-specific expression of Bach1, as observed for other genes on HSA21 and as postulated by the gene dosage hypothesis [102].

Overall, data support that the triplication of genes encoded on HSA21 (e.g., *BACH1*) is involved in the appearance of detrimental conditions, such as increased OS, that over time might contributes to the early development of AD pathology in individuals with DS.

### 3.3. Protein Quality Control Systems

To maintain cell health, proteins must be properly synthesized, folded with high fidelity, assembled, correctly localized, and degraded. Specialized mechanisms respond to malfunction in these essential processes to maintain or re-establish proteostasis, when intracellular signaling networks are triggered by a variety of stress sensor molecules. The UPR senses misfolded protein accumulation in the ER, [2,9,12], whereas the ISR, a central and evolutionarily conserved signaling network, responds to stress conditions from both the lumen of the ER and the cytosol [103] (Figure 1). Indeed, ISR induction can be coupled to UPR activation [104,105]. In the last few years, many efforts have been made to understand how the ISR and the UPR are implicated in the neurodegenerative process of DS. Accumulating evidence support the concept of dysregulated UPR and ISR as key mechanisms, [59,61,62,106,107], which due to their persistent activation could explain the long-term memory and synaptic plasticity deficits in DS. However, how the genetic of DS triggers UPR and ISR still needs to be elucidated.

#### 3.3.1. The Integrated Stress Response

The ISR, a cellular signaling network, couples the detection of cellular stresses to the inhibition of translation initiation. Four different kinases are linked to the ISR sense stress conditions: PERK detects the accumulation of unfolded proteins in the lumen of the ER activating the so-called UPR, protein kinase R (PKR) senses double-stranded RNA, general control nonderepressible 2 (GCN2) responds to amino acid deprivation, and eukaryotic translation initiation factor 2-alpha kinase 1 (EIF2AK1 or HRI) senses heme deficiency. Once activated, these kinases converge on the phosphorylation of the alpha subunit of eukaryotic translation initiation factor 2 (eIF2) at serine 51 to elicit a translational and transcriptional stress response [9]. Phosphorylated eIF2α (p-eIF2α) binds tightly to eIF2β, preventing the formation of the larger complex (eIF2•GTP•methionyl-intiator tRNA ternary complex (TC)), thus inhibiting the initiation of translation, shutting down protein synthesis, and therefore reducing the load of proteins at the ER. As expected, mRNA translation rates are reduced globally as TCs become rate-limiting for translation initiation. Paradoxically, the phosphorylation of eIF2α also triggers the translation of specific mRNAs, including key transcription factors, such as ATF4 [12,108,109,110] and ATF5 [111], or signaling proteins like CHOP [112], GADD34 [113] and in neurons, OPHN1 [114]. These mRNAs contain short inhibitory upstream open reading frames in their 5′-untranslated regions that prevent translation initiation at their canonical AUGs. However, the precise mechanism by which these mRNAs are translationally controlled remains unclear. By tuning down general mRNA translation and upregulating the synthesis of a few proteins that drive a new transcriptional program, the ISR aims to maintain or reestablish physiological homeostasis. In addition to the four specialized kinases that phosphorylate eIF2α, two dedicated phosphatases, GADD34 and the protein phosphatase 1 (PP1), antagonize this reaction. ATF4 activation plays a critical role in cell adaptation or in the activation of apoptosis; this dual activation is based on the extent of its activation. The mechanisms responsible of ATF4 function are crucial to understand the role of the UPR and ISR in cell survival versus death decisions (Figure 1). A key factor regulating the switch from activating pro-survival to cell death pathways is the extent of time the stress persists. Sustained ATF4 levels upregulate pro-apoptotic proteins such as CHOP and growth arrest and DNA damage- inducible 34 (GADD34) [12,115]. ATF4 facilitates the expression of CHOP, which in turn promotes apoptosis by enhancing expression of DR5 and tribbles-related protein 3. The ISR also modulates the two major forms of synaptic plasticity in the mammalian brain, protein synthesis-dependent long-term potentiation (LTP) and long-term depression (LTD) [116,117,118] that are crucial for long-term memory formation. The activation of the ISR has been implicated in a variety of neurodegenerative disorders, including AD and recent evidence pointed out its implications in DS. Costa-Mattioli and colleagues demonstrated the activation of the ISR and confirm phosphorylation of eIF2α, in the hippocampus of a mouse model of DS (Ts65Dn), in post-mortem brain samples from people with DS, and in induced pluripotent stem cells derived from individuals with DS [62]. According to authors the increased levels of p-eIF2α in DS is mediated by the activation of the PKR branch of the ISR [62]. Thus, tuning the activation of ISR emerges as a promising avenue to rescue proteostasis and reverse the cognitive dysfunction in DS.

#### 3.3.2. The Unfolded Protein Response

As mentioned above the UPR is an intracellular signaling pathway that is activated by the buildup of unfolded proteins in the ER. UPR activation triggers an widespread transcriptional response, which corrects the ER protein folding capacity according to its needs [12]. As such, the UPR represents a paradigm of an intracellular control mechanisms that amends organelle abundance in response to environmental or developmental signs. The UPR increases the amount of ER membrane and its components, including chaperones and protein-modifying enzymes, needed to fold proteins. The UPR also reduces the translation and loading of proteins into the ER and improves the targeting of unfolded proteins in the ER for degradation. If homeostatic balance is not restored after UPR induction, i.e., if acute UPR remains induced for a prolonged time, the cell commits apoptosis and the UPR results in a chronic activation [11]. ER stress results from abnormalities that overwhelm normal ER performance such as the blockage of ER protein clearance pathways [19], calcium disruptors, hypoglycemia, exposition to tunicamycin, thapsigargin, dithiothreitol, and hypoxia [119]. In response to ER stress, the cell activates the UPR, whose scope is to reestablish proper ER function by reducing input of nascent proteins and by increasing output of folded proteins [115]. In consequence, the UPR regulates size, shape, and the abundance of luminal and transmembrane proteins [120], all of which contribute to the restoration of homeostasis. The activation of the UPR begins by the dissociation of glucose-regulating proteins (GRPs) from three types of ER transmembrane anchors, namely inositol-requiring protein 1 (IRE1), activating transcription factor 6 (ATF6), or PERK. Once disconnected from the membrane, GRPs associate with nascent proteins to assist their folding and secretion from the ER [121]. The most abundant GRPs are Grp78 (mostly known as BiP) and Grp94 [122]. Meanwhile, each anchor, IRE1, ATF6, and PERK is free to initiate its own signaling pathways (Figure 1). Under homeostatic conditions, IRE1 is constitutively bound to BiP, but once detached from it, IRE1 dimerizes and auto phosphorylates activating RNase domains. Phosphorylated and active IRE1 targets and cleaves X box-binding protein 1 (XBP1), which is a transcriptional activator of UPR controlled genes, which encode for proteins belonging to ER chaperone family or to ERAD pathways [123,124]. Under sustained ER stress, IRE1 mediates the activation of signaling cascades involved in cell death, such as the apoptosis signal- regulated kinase 1 (ASK1) and c-Jun N-terminal kinase (JNK) [125,126,127]. The ATF6 pathway also begins by its dissociation from BiP. The accumulation of improperly folded proteins in the ER causes ATF6 to be exported to the Golgi apparatus and processed by the S1P and S2P proteases [11]. This process mainly leads to the release of the cytosolic fragment domain of ATF6 [11]. In the nucleus, the ATF6 cytosolic domain, simultaneously with XBP1s, upregulates the expression of CHOP and other genes involved in the regulation of ER size, protein-folding capacity, and the ERAD [119,128,129,130]. The third branch of the UPR is initiated by PERK and its overall objective is to reduce translation of mRNA, through the phosphorylation of eIF2α, in order to limit the input of nascent proteins in the ER. Furtherly, PERK is able to target and phosphorylate Nrf2 [131,132] promoting the expression of proteins involved in the adaptation to oxidative stress [133]. Upon activation of the UPR, PERK-directed phosphorylation of Nrf2 dissociates the Keap1/Nrf2 complex favoring Nrf2 translocation to the nucleus where it activates the transcription of antioxidant proteins (Figure 1).

Protein misfolding and aggregation, mitochondrial dysfunction, and oxidative stress are all common features of age-associated neurodegenerative disorders [134,135,136,137,138]. The initial studies on DS patient-derived lymphoblastoid cells (LCLs) and fibroblasts were conducted by Aivazidis and colleagues [106], which demonstrated a constitutive induction of the UPR. DS LCLs showed a modest, but significant up-regulation in the expression of UPR-related genes (CHOP, ATF6, XBP1, PDI, BiP, GRP94, CNE). Meanwhile, both DS LCLs and fibroblasts demonstrated a consistent overexpression of cleaved/activated (50kD fragment) ATF6 protein [106]. The overexpression of ATF6, CHOP and BiP was also demonstrated by our laboratory in PBMCs from DS children, by using an in-depth label-free shotgun proteomics approach, and in relative DS LCLs [59,61]. Intriguingly, DS peripheral cells also demonstrated a close connection between persistent UPR induction and increased OS. In addition, we also demonstrated that DS LCLs, due to sustained ER stress, are vulnerable to cell death when the UPR is further challenged by thapsigargin [61]. In parallel, we also demonstrated an aberrant activation of the UPR in the frontal cortex from DS individuals prior (DSy) and after the development of AD (DS-AD) pathology. Our study delineates a selective activation of PERK along with the increase of eIF2α phosphorylation. Along with the increased of PERK and eIF2α, we also observed the increased expression of the transcription factor ATF4 and CHOP in DSy and DS-AD brains [61]. Interestingly the reduction in GADD34 protein levels in DS and DS-AD suggests the loss of eIF2α normalization and its effective overactivation with the potential consequent translation reduction. The analysis of Ts65Dn frontal cortex [107] supported the contribution of ER stress in DS neuropathology as demonstrated by the consistent and selective activation of the PERK pathway. In particular, the increased expression levels of PERK, eIF2α, ATF4 and CHOP was observed in DS mice at 3 months of age. This study proposed that chronic PERK activation was an early and toxic mechanism in DS, which precedes tau and Aβ accumulation and might be associated with increased OS [107]. Further, data collected in Ts65Dn mice were corroborated on the Ts2Cje model of DS that reported a comparable early alteration of the PERK pathway [61]. Studies conducted in DS human and mouse brain suggest a putative role for the trisomic-related dysregulation of gene expression in the observed chronic UPR induction, however, the role of ER stress in DS neuropathology and how DS genetics may alter the UPR is far from being understood.

### 3.4. Protein Degradation Pathways

Among the putative mechanisms proposed to be involved in DS neuropathology, defects in protein degradation have emerged as a prominent mechanism triggering neurodegeneration [8,58,106,139] (Figure 1). UPS and autophagy represent two principal pathways of protein and organelle clearance in eukaryotic cells [2,7,9,10]. The significance of protein folding, surveillance, and degradation systems in neurons is evident since post mitotic cells rely on the proteostasis network to cope with normal and damaged proteins and to maintain its operation. The failure of neuronal proteostasis, due to the alteration of PQC systems, could support the aggregation of disease-specific toxic proteins, that might, directly or indirectly, target or interfere with different biological components of the cell [7,22,23,140,141,142]. In turn, the formation of misfolded/unfolded protein aggregates may lead to the impairment of degradative systems, exacerbating toxic protein deposition and resulting in ER stress and oxidative damage [7,10,141].

#### 3.4.1. The Ubiquitin Proteasome System

The proteasomal system is located in the cytosol and in the nucleus, and it is responsible for the degradation of more than 70–80% of intracellular proteins. The UPS degrades misfolded, oxidized, or damaged proteins, but it is also involved in removing proteins from many cellular processes, such as signal transduction, cell cycle regulation, and cell death; furthermore, it ultimately regulates gene transcription [143,144,145]. The majority of the proteins are directed to proteasomal degradation after being covalently modified with ubiquitin through the formation of an isopeptide bond between. This conjugation normally involves three types of enzyme: E1 hydrolyzes ATP and forms a thioester-linked conjugate between itself and ubiquitin; E2 receives ubiquitin from E1 and forms a thioester intermediate with ubiquitin; and E3 binds both E2 and the substrate and transfers the ubiquitin to the substrate [146,147,148]. In some circumstances, a fourth ubiquitination enzyme, E4, is necessary to extend a polyubiquitin chain [149]. Polyubiquitin chain then is recognized by the proteasome, a multicatalytic complex indicated as the 26S proteasome. Three proteolytic activities of the proteasome are recognized and are the chymotrypsin-like, caspase-like, and trypsin- like [146,147,148,150]. It has been suggested that the oxidation of proteins causes the exposure of hydrophobic moieties to the surface via partial unfolding that are targeted by proteasome [150,151,152].

An analysis of proteasome degradative functionality in DS human frontal cortex demonstrated a decrease in trypsin-like, chymotrypsin-like, and caspase-like activities supporting an impairment of protein clearance during the early stages of the disease [8]. To note, the UPS and its caspase-like activity, by being able to regulate the degradation of pro-apoptotic molecules, holds a key complex role in the activation of apoptosis [153]. However, despite many authors described the increase of apoptotic markers in the DS brain, the role of such programmed cell-death process has not been fully elucidated [154,155]. Among the altered mechanisms leading to reduced proteasome functionality, proteomics studies from our laboratory described aberrant post-translational regulation of UCH-L1 [8,79]. UCH-L1 hydrolyzes ubiquitin (Ub) chains from the carboxyl terminus allowing the degraded protein to gain access to the proteasome [156,157]. UCH-L1, a major target of oxidative damage in DS brain undergoes aberrant poly-ubiquitinylation, suggesting its irreversible structural impairment, loss of activity and a possible target for degradation [8,79,158]. UCH-L1 has been shown to be carbonylated in AD brains and this event was associated with the loss of its ubiquitin hydrolase and/or ligase activity [159,160,161,162]. Thus, UCH-L1 oxidation might lead to the dysfunction of the neuronal ubiquitination/de-ubiquitination machinery, to the accumulation of damaged proteins and to the formation of protein aggregates. In agreement with the alteration of proteasome activity and with the aberrant ubiquitination/deubiquitination process, the accumulation of polyubiquitinated proteins is observed in the brain of DS individuals before and after the development of AD [158]. Indeed, unfolded/misfolded proteins might be retargeted by Ub for degradation but then maintained in the poly-ubiquitinated form for degradation and the ubiquitin recycling steps of the UPS. In agreement, studies by Aivazidis and colleagues also observed increased levels of polyubiquitinated proteins in DS fibroblast associated with significantly reduced chymotrypsin-like and trypsin-like proteolytic activity [106]. Proteasome chymotrypsin-like proteolytic activity was reduced in the cerebellum of Ts65Dn mice in comparison with euploid animals and ubiquitin immunohistochemistry showed an increase in ubiquitinated proteins [163].

By sorting genes that map on chromosome 21, a genetic link between trisomy and aberrant UPS function may emerge. Indeed, a number of HSA21 genes including, ubiquitin specific peptidase 25 (USP25), ubiquitin specific peptidase 16 (USP16), proteasome assembly chaperone 1 (PSMG1), ubiquitin associated and SH3 domain containing A (UBASH3A), ubiquitin conjugating enzyme E2 G2 (UBE2G2), and listerin E3 ubiquitin protein ligase 1 (LTN1), encode for proteins that hold a role in UPS clearance activity and might be implicated in the formation of toxic aggregates [164,165]. However, studies aimed to decipher the direct association between the triplication of genes belonging to UPS and aberrant proteostasis in DS brain, have not been conducted yet. Notably, the increased oxidation of SOD1 was found in the cortex of transgenic mice expressing h-SOD1, authors demonstrated that increased oxidation of SOD1, a condition observed in DS human brain might be involved in the inhibition of proteasome activity [166].

#### 3.4.2. Autophagy

Autophagy is a cellular mechanism that removes degraded/dysfunctional components and plays a key role in cell survival and in preserving cell metabolic balance [142]. Whereas the UPS degrades mainly short-lived proteins, autophagy is specialized in the removal of long-lived proteins, and unlike the UPS, is uniquely able to degrade whole organelles such as mitochondria, peroxisomes, and the ER [140]. The homeostatic role of autophagy includes both nonselective and selective degradation mechanisms. Nonselective degradation is involved in the basal turnover of cytoplasmic components, while selective degradation is involved in targeting damaged or aggregated organelles and proteins, thus operating as an indispensable cellular quality-control function [167]. Autophagy is considered as a recovery process that provides essential components to sustain principal metabolic functions during starvation or stress [168]. There are three principal routes of autophagic degradation, which differ mainly in the manners of cargo delivery to the lysosome: macroautophagy, microautophagy, and chaperone-mediated autophagy (CMA) [6,22]. During macroautophagy, bulk cytoplasmic components are sequestered in a double-membrane structure known as autophagosome, which is successively trafficked to the lysosome. The autophagosome outer membrane then fuses to the lysosome, leading to degradation. In microautophagy and CMA, cargo is directly taken up by the lysosome, either through the invagination of the lysosomal membrane or by unfolding and translocation of proteins with a specific signal sequence that is recognized by the LAMP2A receptor on the lysosome [142]. Among the three type of autophagy, macroautophagy is the best characterized and researchers often refer to it simply as autophagy. This process is controlled primarily by two crucial signaling proteins: the mammalian target of rapamycin (mTOR) and the AMP- activated protein kinase (AMPK) [169]. Under ordinary nutrient circumstances, active mTOR complex 1 (mTORC1) phosphorylates Ulk1 and sequesters it in a complex with Atg13 and FIP200, thereby inhibiting autophagy. Starvation, amino acid deprivation, or growth factors remove mTOR restraint allowing Ulk1 to promote autophagy. AMPK is a major positive regulator of autophagy, which is activated by high AMP/ATP ratio. Under low intracellular energy, activated AMPK induces autophagy by Ulk1 activation and by mTORC1 inhibition via phosphorylation of Raptor. Both AMPK and mTOR also control cell growth and metabolism, coupling autophagy to these processes [169]. Once activated, Ulk1 initiate autophagosome nucleation by creating the class III phosphatidylinositol 3-kinase (PtdIns3K) complex [22]. PtdIns3k, together with other Atg-proteins, has the function to recruit two ubiquitin-like conjugation systems, which are necessary for the expansion and elongation of the phagophore and with the formation of mature autophagosomes: Atg12–Atg5-Atg16L1 and the light-chain 3 (LC3) system [170]. At the last, autophagosome fuses with the lysosome forming the autophagolysosome where the cargo degradation is determined by the acidic environment of the lysosome, which is maintained by the activity of the V_0_-ATPase proton pump. Lysosomal hydrolases such as cathepsin B, D, and L are involved in the cleavage of autophagic substrates, while the resulting molecules are transported back to the cytosol for nutrient recycling [171]. A connection between the ER and autophagy was recently proposed to occur through the IP3 receptor and BCL-2 [141]. Further, many laboratories have shown that ER stress triggers autophagy, and this effect is also regulated by UPR stress sensors such as IRE1 and PERK [172,173]. Thus, autophagy may also serve as a mechanism to eliminate portions of damaged ER under stress conditions or to control the rate of ER expansion [174].

Defective autophagy has been observed during aging and neurodegenerative disorders supporting its fundamental role in advancing the progression of brain damage and cognitive decline [5,6,22,23,57,142,175,176,177]. Consistently, studies using transgenic models have highlighted the crucial role of constitutive autophagy in protecting neurons [25,178]. Recent studies from our laboratory employed the frontal cortex from young DS autopsy cases and from DS subjects with AD neuropathology to confirm that aberrant mTOR/autophagy is an early degenerating event in the brain that contributes to acceleration of AD hallmarks and to the development of AD-like cognitive decline [8,24,139]. Our results showed that the hyperactivation of the PI3K/AKT/mTOR axis was associated with decreased autophagosome formation and increased levels of Aβ and p-tau [139]. In particular, we demonstrated in DS brain the increase of mTORC1 phosphorylation, as a result of the aberrant regulation of the insulin signaling, which impinge on autophagy induction by reducing autophagosome formation as indexed by LC3II/I levels [139]. In parallel, Iyer and colleagues analyzed the expression patterns and cellular distribution of the components of the mTORC1 pathway in human hippocampi of DS subjects during prenatal and early postnatal development, and in the presence of AD pathology [179]. The study showed the prenatal upregulation of pS6 and p70S6K, that persisted throughout postnatal development, while the upregulation of p4E-BP1 and mTOR was detected postnatally in DS hippocampi [179]. This study also confirmed the upregulation of mTORC1 components and downstream signals in DS-AD patients, showing a positive correlation with total tau and p-tau [179]. Evidence by the Nixon’s group confirmed a strong mTOR hyperactivation in primary human fibroblast from DS subjects which globally suppresses macroautophagy induction [60].

Furthermore, autophagy-deficient fibroblast demonstrated the accumulation of damaged mitochondria with a consequent increase in oxidative stress. This finding was associated with the reduced activation of the mitophagy pathway as regard as dysregulated PINK1/PARKIN signal [60]. Studies on the human brain from DS cases also proposed that increased ROS can target autophagy and exacerbate the inhibition of degradation pathways [7]. Studies from our laboratory reported the increase oxidation of several components of the autophagy machinery supporting the link between aberrant mTOR/autophagy, altered proteostasis network and increased OS [8,79]. Specifically, our results showed that oxidative damage targets, among others, V_0_-ATPase, cathepsin D, and GFAP and this was coupled with a decreased LC3 II/I ratio early in the brain of individuals with DS and DS with AD [8,79]. V_0_-ATPase pump is essential for acidic lysosomal pH and the mutation of the lysosomal ATPase genes are a well-recognized risk factor for autophagy related neurodegenerative diseases. Thus, oxidized V_0_-ATPase might have an altered ability to regulate intracellular pH thus affecting proper lysosome functionality [180,181]. In addition, a recent report showed that V_0_-ATPase is necessary for amino acids to activate mTORC1, suggesting that V_0_-ATPase is an active component of the mTOR pathway [182]. A study using primary human DS fibroblasts reported the early dysfunction in lysosomal degradative capacity that was dependent on the additional copy of the APP gene and, more specifically, on the C99 APP carboxyl terminal fragment. Nixon and colleagues found that a moderate increase in C99 levels was sufficient to impair lysosomal function in DS due to an increase in the luminal organelle pH [183]. Noteworthy, this effect was mediated by a direct physical interaction between C99 with the cytosol exposed domain of V_0_-ATPase, which was reverted by lowering C99 levels or adding acidic nanoparticles [183]. Interestingly it was shown that GFAP is an important regulator of CMA and it was proposed to interact at the lysosomal membrane either with LAMP-2A or with the elongation factor 1α [184]. Furthermore, CatD, which is normally localized within lysosomes and participates in the degradative processes, was found to be oxidized in DS but resulted in a slight increase of enzyme activity [8]. The activation of CatD, suggest the occurrence of a compensatory response to partially disturbed autophagy and to the accumulation of toxic aggregates [24]. The alteration of mTOR/autophagy axis was also observed in different mouse model of DS. In Ts1Cje mice the hyperactivation of Akt-mTOR pathway was demonstrated in dendrites of hippocampal neurons [185]. The authors showed that the levels of p-Akt, p-mTOR (Ser-2448), p-p70S6K (Thr-389), p-S6 (Ser-235/236), and p-4EBP1 (Ser-65) were increased approximately 2-fold in dendrites of Ts1Cje- derived hippocampal neurons [185]. Further, mTOR signaling is deregulated in the brain of the Tc1 mouse model of DS [186]. Recent studies from our laboratory demonstrated that 9 months old Ts65Dn brain recapitulates the aberrant regulation of the AKT/mTOR/autophagy associated with the accumulation of Aβ and p-Tau [63,187]. Here also we observed that the reduction of autophagy, demonstrated by altered LC3 II/I, Atg12/5 and Atg7 levels, contributed to the increase of oxidative stress, to defective UPS and to the alteration of early synaptic proteins in Ts65Dn mice [63,187]. Similarly, the alteration of autophagy was also observed in Ts2Cje mice [56].

Endosomal pathology has been also shown in human DS neurons, in neurons of DS mouse models, and in human DS fibroblasts [188,189,190,191,192]. Several evidence and emerging hypotheses suggest that triplicated genes, such as ITSN1 and SYNJ1, are implicated in triggering the abnormalities of endolysosomal pathway [193]. Higher exosome levels were found in frontal cortices of subject with DS and in the brains of two DS mouse models, Ts2Cje and Ts65Dn [194].

## 4. Targeting Stress Responses in Down Syndrome Brain

### 4.1. Antioxidant Molecules

The use of antioxidant molecules in the context of DS have gained a great deal of attention in the last decade. A number of studies have been conducted in in vitro and in vivo models of DS with the aim of reducing the early increase of OS. Furthermore, compounds with well-known antioxidant properties have been tested in clinical trials with DS subjects demonstrating promising efficacy in ameliorating cognitive defects and a general safety use (Table 1). Despite the significance of the administration of antioxidant molecules in DS, clinical studies conducted so far have been mostly unsatisfying in respect to pre-clinical ones for a number of reasons that involves: (i) the type of combinations of antioxidants used; (ii) the dosage of the antioxidants administered; (iii) the small sample sizes employed for human and mouse studies; (iv) the genetic difference between the DS models used; and (v) the genetic variability of DS subjects [71].

Dietary supplementation of Ts65Dn mice with α-tocopherol (50 mg/Kg/day) for 5 months was shown to reduce OS, attenuates cholinergic neuron degeneration, preserve hippocampal morphology, and improves spatial working memory [195]. Further, it was shown that α-Tocopherol acetate (0.1% *w*/*w* for Kg of diet) administered to pregnant Ts65Dn mice, from the day of conception throughout the pregnancy and administered to pups from birth to the end of the behavioral testing period mitigated cognitive deficiencies, reduced levels of lipid peroxidation and improved hypocellularity in the hippocampal dentate gyrus [196]. In a randomized, double-blind, placebo-controlled trial, Lott et al. [197] daily administered α-tocopherol (900 IU), ascorbic acid (200 mg), and α-lipoic acid (600 mg) to individuals with DS and AD for two years but no cognitive improvement was observed [198]. In a further study, adults with DS over 50 years received vitamin E orally (1000 IU/twice daily) for over 3 years demonstrated that the treatment did not delay the cognitive decline observed in DS [199]. In a randomized controlled trial, Mustafa and colleagues [200] evaluated the ability of vitamin E (266 mg/day) and α-lipoic acid (100 mg/day) administered for 4-month period to children and teenagers with DS ranging from 7 to 15 years of age. α-tocopherol exerted a mild decrease of oxidative stress at the DNA level in children with DS. In another study, daily antioxidant treatment with a combination of vitamins E (400 mg/day) and C (500 mg/day) given to children and teenagers with DS over a 6-month period decreased the blood level of lipid peroxidation [201]. In a later study, the same group also demonstrated that the administration of this combined antioxidant treatment attenuated systemic oxidative damage [202]. Tiano and colleagues in 2012 supplemented children with DS with coenzyme Q10 (4 mg/kg/day) for 6 or 20 months. In the younger age group (5–12 years) coenzyme Q10 inhibited oxidative damage to DNA pyrimidines and in the aged group of 13–17 years oxidized purines were reduced [203]. However, a 4-year long diet supplementation of coenzyme Q10 in children with DS did not affect RNA or DNA oxidation [204]. Melatonin exerts various antioxidant effects, including being a potent ROS scavenger, regulating anti- and pro-oxidant enzymes, and stimulating the rescue of oxidized molecules [205]. Melatonin (0.5 mg/day in water) was administered for 5 months to 5–6-month-old Ts65Dn mice. Melatonin improved spatial learning and memory, rescued the impairment of adult neurogenesis, decreased hippocampal granule cells density, and reduced synaptic inhibition in trisomic mice [206,207,208]. In addition, melatonin decreased the levels of lipid peroxidation in the hippocampus of Ts65Dn mice but did not significantly reduced Aβ levels. Apigenin is a small molecule approved by the FDA for its antioxidant, anti-inflammatory, and anti-apoptotic properties. Apigenin reduced oxidative stress and improved total antioxidant capacity in amniocytes derived from second-trimester fetuses with T21. Further, apigenin (200-250 mg/Kg/day in chow) given to pregnant Ts1Cje mothers and to their pups up to 8-10 weeks of postnatal life, improved postnatal behavioral including spatial olfactory memory [209]. Interestingly, this study reported sex-specific effects on exploratory behavior and long-term hippocampal memory in adult Ts1Cje mice, with males showing significantly more improvement than females. Gene expression and protein level analyses shown that apigenin had a pleiotropic action and reached its therapeutic efficacy partially through suppression of pro-inflammatory responses and NFkB signaling, and by the induction of anti-inflammatory response [209]. Epigallocatechin gallate (EGCG), the most common catechin present in green tea, promote neuroprotection by different mechanisms, including its antioxidant actions and its effects on molecular pathways implicated in the maintenance of mitochondrial homeostasis [71]. EGCG is a specific inhibitor of the kinase activity of the chromosome 21-encoded DYRK1A, which is involved in brain development, and the modulation of synaptic plasticity [91]. EGCG has been tested as a therapeutic agent in a murine model of DS and in clinical trials showing promising results for the treatment of brain pathology [71]. In 2013, Valenti and colleagues demonstrated the ability of EGCG (20 μM) to reduce oxidative stress and mitochondrial energy deficit through a mechanism involving cAMP/PKA and Sirt1 signaling pathways in human DS cell cultures [210]. The in vivo oral administration of EGCG to Ts65Dn mice (2–3 mg/day), by de la Torre et. al, showed to efficiently rescue cognition by improving hippocampal-dependent learning deficits [211]. Subsequently, Souchet and colleagues delivered commercially available EGCG-containing extracts (Polyphenon 60 at 225 mg/kg/day) in water to Ts65dn mice for 4 weeks and this treatment was able to restore excitatory/inhibitory (E/I) imbalance through the modulation of the GABA pathway [212]. In a further study, Stagni et al. treated Ts65Dn mice with EGCG (25 mg/Kg/day) by subcutaneous injection from 3 to 15 postnatal days [64]. The effects of the treatment were observed at two different time points: at 15 postnatal days and at 45 postnatal days. Intriguingly, the authors demonstrated that at 15 postnatal days trisomic pups undergoing EGCG treatment exhibited the restoration of neurogenesis and of total hippocampal granule cells number, further the recovery of pre- and postsynaptic protein levels was observed in the dentate gyrus (DG), hippocampus and neocortex. However, after 1 month the beneficial outcomes were lost [64]. In 2016 Catuara-Solarz and colleagues explored the effects of a combined therapy with environmental enrichment (EE) and green tea extract containing 45% of EGCG (30 mg/kg per day in drinking water) in young Ts65Dn for 30 days [213]. Such treatment was able to rescue dendritic spine density of CA1 region of the hippocampus and normalize the proportion of E/I synaptic markers in CA1 and dentate gyrus, thus improving corticohippocampal-dependent learning and memory. Prenatal treatment of the Dp(16)1Yey mouse model, demonstrated that the administration of EGCG at a final daily dose of 50 mg/kg in food pellets reduced levels of inhibitory markers, restored VGAT1/VGLUT1 balance, and rescued density of GAD67 interneurons, finally resulting in improved novel object recognition memory [214]. In a phase I randomized controlled clinical trial, de la Torre and colleagues demonstrated that a 6 months-long treatment of EGCG (9 mg/kg/day) to young adults with DS was safe, and reduced plasma homocysteine and had a mild positive effect on cognitive performances [211]. The following double-blind, placebo-controlled, phase II trial conducted on young adults with DS treated with EGCG (9 mg/kg/day) for 12 months indicate that EGCG had beneficial effects on memory and executive deficits with enhancement of the everyday life competence in young adults with Down’s syndrome, although some domains were not significantly modified by the treatment [215].

### 4.2. ISR and UPR Inhibitors

The pharmacological modulation of ISR and UPR is emerging as a promising avenue to alleviate the cognitive decline resulting from a disruption in protein homeostasis in DS (Table 1). The inhibition of a PKR with a PKRi rescued the deficits in long-term memory and synaptic plasticity in Ts65Dn mice [216]. PKR in Ts65Dn was found to be inhibited also by daily subcutaneous injection from P3 to P15 of fluoxetine (5 mg/kg from P3 to P7; 10 mg/kg from P8 to P15), a selective serotonin reuptake inhibitor. This treatment improved long-term memory in Ts65Dn mice [217,218]. In the past year, a study conducted by Zhu et al. [62] demonstrated that the pharmacological inhibition of the ISR restore protein synthesis and improve long-term memory in Ts65Dn mice. Ts65Dn mice were treated with the small-molecule, drug-like ISR inhibitor ISRIB [62]. ISRIB is a potent eIF2B activator that enhances GEF activity by facilitating eIF2B assembly into its decameric holoenzyme, resulting in the reversal of p-eIF2α translation repression [219,220]. The pharmacological suppression of the ISR, by inhibiting the ISR-inducing double-stranded RNA-activated protein kinase or boosted the function of eIF2:eIF2B complex, reversed the changes in translation and inhibitory synaptic transmission and rescued the synaptic plasticity and long-term memory deficits seen in DS mice. In our recent study, the pharmacological reduction of the PERK pathway of the UPR in DS was found to restore protein synthesis and to provide positive outcomes in Ts2Cje mice. By treating Ts2Cje mice with GSK2606414, a well-established PERK inhibitor [221,222], we observed that the pharmacological decrease of chronic PERK induction was able to restore downstream signals, rescue dysfunctional proteostasis, and reduce oxidative stress [61]. In particular, the intranasal treatment with GSK2606414 in DS mice significantly reduced cortical PERK and eIF2α activation, which in turn rescued protein translation demonstrated by in vivo puromycin administration [61]. Further, pharmacological inhibition of PERK reduced protein oxidation by the induction of Nrf2 related antioxidant response thus allowing the transcription of HO-1 and NQO1 antioxidant genes, thus conceivably contributing to the reduction of oxidative stress levels [61]. Intriguingly, the treatment with GSK2606414 promote proper Bach1 degradation, enhancing the antioxidant response, through the binding of Nrf2 to the ARE. Therefore, the rescue of PERK, by recovering of Nrf2/Bach1 balance was able to remove the disengagement between PERK and Nrf2 and protect the DS brain from increased OS.

### 4.3. Autophagy Inducers

A number of pharmacological treatments targeting the Akt/mTOR signaling rescued the induction of autophagy thus ameliorating the reduction of protein degradation observed in DS (Table 1). In 2015, Montesinos’s laboratory administered rapamycin (10 mg/kg) to a Ts1Cje mouse model, previously investigated for Akt–mTOR signaling, by i.p. injection for 5 days showed that the significant impairment of spatial long-term memory persistence, investigated by Barnes maze, was restored by rapamycin [223]. Although specific markers of autophagy were investigated the rescue of mTOR pathway suggested the involvement of autophagy in the improvement of cognitive performances. Subsequently, in our laboratory, we administered rapamycin (1μg/3 times per week) by intranasal delivery to 6 months old Ts65Dn for 12 weeks [188]. The treatment demonstrated a significant effect on cognitive performance in Ts65Dn mice as indexed by performance of radial arm maze (RAM) and novel object recognition (NOR) tests. In particular, the RAM test revealed improvement of reference and working memory, while NOR test showed improvement after rapamycin administration. The analysis of mTOR signaling, after rapamycin delivery, demonstrated the inhibition of mTOR in the hippocampus, which led to the rescued induction of autophagy as indexed by LC3 II/I and Atg12-Atg5 complex levels [188]. Further, the recovery of mTOR/autophagy axis was associated with improved insulin signaling, reduced APP levels, APP processing, APP metabolites production and tau hyperphosphorylation. In a following study we demonstrated that a decrease of protein-bound HNE levels improved arginase-1 and protein phosphatase 2A activity [63]. Lately, we demonstrated that rescuing protein O-GlcNAcylation levels in Ts2Cje mice by intranasal Thiamet G administration (25μg/mouse; 2 times/day for 5 days) boosted autophagy induction favoring the restoration of proteostasis [56]. Alldred and colleagues in 2019 examined the impact of perinatal maternal choline supplementation in Ts65Dn mouse to discern the effects on gene expression within adult offspring at ~6 and 11 months of age [224]. The authors found that maternal choline supplementation (5.0 g/kg choline chloride) diet produced significant changes in offspring gene expression levels associated with age-related cognitive decline including the endosomal-lysosomal pathway and autophagy. In particular among the proteins involved in the endosomal-lysosomal and autophagy pathway, autophagy-related 4B cysteine peptidase, cathepsin B, lysosomal-associated membrane protein 2 (Lamp2), myosin VB, palmitoyl-protein thioesterase 1 and RAB27 were found [224]. Recently, a study by Bordi et al. confirmed a strong mTOR hyperactivation associated with PINK1/PARKIN impairment in primary human fibroblast from DS subjects that globally suppresses macroautophagy induction and the expression of proteins critical for autophagosome formation such as ATG7, ATG3 and FOXO1. The inhibition of mTORC1/2 by AZD8055 (0.1 μM) restored autophagy flux, PARKIN/PINK initiation of mitophagy, and the clearance of damaged mitochondria by mitophagy [60].

## 5. Conclusions

In the last two decades, numerous studies have been focused on highlighting the molecular mechanisms promoting AD neuropathology in DS [17,36,57,88,198]. In particular, it was demonstrated that the trisomy of HSA21 is highly associated with redox imbalance and altered protein homeostasis in brain cells. These events, being either the direct result of gene triplication or the indirect effect of genome instability, represent early signatures of the neurodegenerative process, as reported also in AD. The redox buffer of the brain is preserved by the induction of antioxidant responses, which detoxify ROS and protect biological components in the CNS from oxidation and from the alteration of their functional status [71,198]. In DS, the altered expression ratio between SOD1 and CAT/GPX, the reduced induction of the Nrf2 antioxidant response, as results of triplicated Bach1, and the observed mitochondrial defects promote the increase of OS leading to a critical pro-oxidant environment that result in massive protein oxidation. In turn, pathways involved in proteostasis surveillance and conservation, including ISR, UPR, UPS, and autophagy, display altered regulatory mechanisms favoring the build-up of unfolded/misfolded proteins and of toxic protein aggregates [7,61,62,63,106,139]. Within this picture, the reduction of brain antioxidant capability and the impairment of protein synthesis/folding/clearance mechanisms represent major critical events, associated with trisomy 21, that initiate a self-sustained vicious cycle of degenerative processes, which may promote and contribute to the progression of APP- and tau-related pathological hallmarks and to the development of cognitive decline [7,10]. A number of pre-clinical and clinical studies have been applied in DS with the aim of rescuing either redox imbalance or aberrant proteostasis. Initially, the beneficial effects of antioxidant molecules were tested in DS mouse models and demonstrated high efficacy in reducing oxidative damage and in reverting DS phenotype. However, clinical trials, despite being promising and well tolerated, exerted only modest effects on cognitive performances in DS individuals, for a number of reasons including differences of drugs combination and dosage, sample size and the genetic variability of DS humans and mice employed [71]. In the last decade, a great deal of attention has been also dedicated to the rescue of proteostasis by targeting molecules at the nodes of protein synthesis/degradation processes. Preclinical studies in DS mouse models have indicated the ability of compounds targeting components of the UPR, ISR or autophagy in reducing cell stress, ameliorate pathological hallmarks and improve cognition [61,62,63].

Nevertheless, these molecules have not been tested in DS clinical trials, perhaps due to a need for great insight on the role of PQC systems and protein degradation more studies are needed to address this point. Intriguingly, pre-clinical studies in DS with antioxidant or protein synthesis/folding/degradation related molecules, highlighted the notion that different therapeutic approached ended up in similar outcomes concerning the amelioration of DS neuropathology. On one hand, these observations strengthen the deleterious mutual association between OS and the disruption of PQC/clearance systems; on the other hand, they suggest that therapeutic molecules aimed to reverse redox imbalance and/or aberrant proteostasis reverse or slow this cellular death spiral. Overall, the search for effective therapeutic agents to reduce DS neurodegeneration is a battle far from being resolved, however the rescue of brain redox and protein homeostasis, by arming stress response mechanisms, represent a promising and strategic approach in the fight against the pathobiology and cognitive decline in DS.

## Figures and Tables

**Table 1:** List of all the compounds used in DS neuropathology in clinical and preclinical studies acting on the Unfolded and Integrated stress Response, autophagy and ROS.

Compound	Structure	Target	Study Type	Dosage	Length of the Treatment	Administration Route	Model	Ref.	Outcomes
**Unfolded Protein Response and integrated stress Response inhibitors**									
GSK2606414	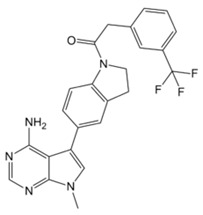	PERK	Preclinical study	0.1 μg/μL	5 days (1× day)	intranasal treatment	Ts2Cje	[61]	Restored protein synthesis; reduced OS
ISRIB	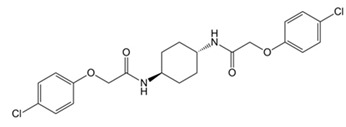	eiF2a	Preclinical study	2.5 mg/kg	7 days (once every 2 days)	i.p. injection	Ts65dn	[62]	Restored protein synthesis; improved long-term memory
PKRi	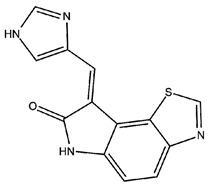	PKR	Preclinical study	0.1 mg/kg	6 days (1× day)	i.p. injection	Ts65dn	[216]	Rescued long-term memory and synaptic plasticity
**Autophagy inducers**									
Rapamycin	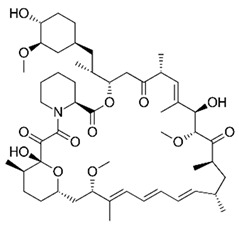	mTOR	Preclinical study	10 mg/kg	5 days	i.p. injection	Ts1Cje	[223]	Restored spatial long-term memory
		mTOR	Preclinical study	1μg	90 days (1× day, 3× week)	intranasal treatment	Ts65dn	[63,187]	Restored autophagy; rescued cognitive performances, reduced AD hallmarks and OS
Thiamet G	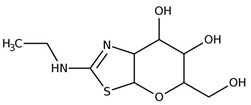	O-GlcNAcase	Preclinical study	25 ug	5 days (2× day)	intranasal treatment	Ts2Cje	[56]	Restored proteostasis; rescued autophagy
Choline	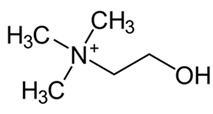	phospholipids/ neurotransmitter	Preclinical study	5.0 g/kg choline chloride	≃ 21 days	maternal supplementation	Ts65dn	[224]	Restored autophagy and endosomal pathway; improved cognition
AZD8055	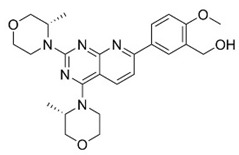	mTORC1/2	Preclinical study	0.1 μM	2, 4 and 8 h	cell treatment	Human fibroblast	[60]	Rescued autophagy; rescued mitophagy
**Antioxidant molecules**									
α-tocopherol (vitamin E)	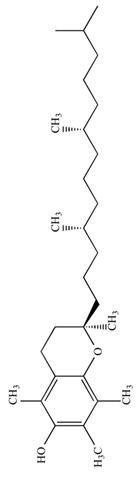	ROS	Preclinical study	50 mg/Kg	5 months	diet supplementation	Ts65dn	[195]	Reduced OS; improved spatial working memory
		ROS	Preclinical study	0.1% *w*/*w* for Kg of diet	Pregnancy and pups	maternal supplementation	Ts65dn	[196]	Improved cognition; reduced lipid peroxidation
		ROS	Randomized, double-blind, placebo-controlled trial	900 IU+ ascorbic acid (200 mg) + α-lipoic acid (600 mg)	2 years (daily)	oral	DS and AD individuals	[197]	No cognition improvement
		ROS	Randomized, placebo-controlled clinical trial	1000 IU	over 3 years (twice daily)	oral	DS over 50 years	[199]	No cognitive improvement
		ROS	randomized controlled trial	266 mg + α-lipoic acid (100 mg/day)	4-months (daily)	oral	DS children	[200]	Reduced OS at DNA level
		ROS	Clinical study	400 mg + Vitamin C (500 mg/day)	over 6 months (daily)	oral	DS children and teenagers	[201]	Reduced blood levels of lipid peroxidation
Coenzyme Q10	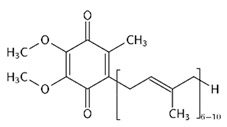	ROS	Clinical study	4 mg/kg	6 or 20 months (daily)	oral	DS children	[203]	Reduced OS at DNA level
		ROS	Clinical study	4 mg/kg	4-year (daily)	oral	DS children	[204]	No reduced OS at RNA or DNA level
Melatonin	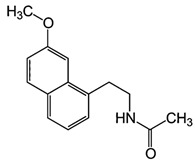	ROS	Preclinical study	(0.5 mg/day)	5 months	water supplementation	Ts65dn	[205,206,207,208]	Improved spatial learning and memory; rescued neurogenesis
Apigenin	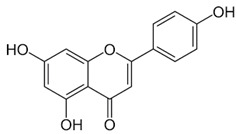	ROS	Preclinical study	333–400 mg/kg	≃ 45 days (pregnancy and lactation)	maternal supplementation	Ts1Cje	[209]	Improved postnatal behavior
EGCG	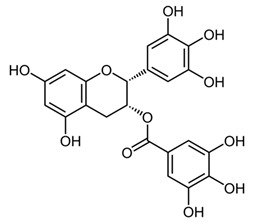	DYRK1A; ROS	Preclinical study	20 μM	72 h (changed every 24 h)	cells treatment	Human DS cell cultures	[210]	Reduced OS and mitochondrial energy deficit
			Preclinical study	2–3 mg/day	1 month	water supplementation	Ts65Dn/TgDyrk1A	[211]	Improved cognition
			Preclinical study	225 mg/kg/day	4 weeks	water supplementation	Ts65Dn	[212]	Restored excitatory/inhibitory (E/I) imbalance (GABA modulation)
			Preclinical study	25 mg/Kg/day	P3 to P15	subcutaneous injection	Ts65Dn	[64]	Restored neurogenesis at P15; no cognitive improvement at P45
			Preclinical study	30 mg/kg/ day	30 days	water supplementation	Ts65Dn	[213]	Rescued CA1 dendritic spine density, improved cognition
			Preclinical study	50 mg/kg	T1 (21 days) T2 (mating until 90 days) T3(P60-P90)	diet supplementation	Dp(16)1Yey	[214]	Rescued GAD67; restored VGAT1/VGLUT1 balance; improved novel object recognition memory
			phase I randomized controlled clinical trials	9 mg/kg/day	6 months	diet supplementation	Young adults with DS	[211]	Reduced plasma homocysteine; rescued cognitive performances
			double-blind, placebo-controlled, phase II trial	9 mg/kg/day	12 months	diet supplementation	Young adults with DS	[215]	Improvement in adaptive behavior

## Data Availability

Not applicable.

## Abbreviations

8-OHdG	8-hydroxy-2’-deoxyguanosine
Aβ	amyloid β
AD	Alzheimer disease
ALS	amyotrophic lateral sclerosis
AMPK	AMP- activated protein kinase
AOR	antioxidant response
APP	amyloid Precursor Protein
ARE	antioxidant response elements
ASK1	apoptosis signal-regulated kinase 1
ATF	cyclic AMP-dependent transcription factor-
ATP	adenosine triphosphate
ATG	autophagy-related gene
Bach1	BTB and CNC homology 1
BCL-2	B-cell lymphoma 2
BiP	immunoglobulin heavy chain-binding protein
CAT	catalase
CatD	cathepsin D
CHOP	C-EBP-Homologous Protein
CMA	chaperone mediated autophagy
DS	Down syndrome
DYRK1A	dual specificity tyrosine phosphorylation regulated kinase 1A
EGCG	epigallocatechin gallate
eIF2	eukaryotic initiation factor 2
ER	endoplasmic reticulum
ERAD	ER associated degradation
GADD	growth arrest DNA damage-inducible transcript
GCN2	general control nonderepressible 2
GFAP	glial fibrillary acidic protein
GPX	glutathione peroxidase
GRP	glucose-regulated protein
HD	Huntington disease
HO-1	heme oxygenase 1
HNE	4-hydroxy-2-nonenals
HSA21	homo sapiens chromosome 21
IRE1	inositol-requiring enzyme 1
IRS1	insulin receptor substrate1
IR	insulin receptor
ISR	integrated stress response
ISRIB	integrated stress response inhibitor
LTP	long-term potentiation
LTD	long-term depression
mTOR	mammalian target of rapamycin
mTORC	mammalian target of rapamycin complex
OGA	O-GlcNAcase
OXPHOS	oxidative phosphorylation
OPHN1	Oligophrenin-1
OS	oxidative stress
JNK	c-Jun N-terminal kinase
Mmu	Mouse chromosome
NDD	neurodegenerative diseases
NFTs	neurofibrillary tangles
NQO1	NADPH quinone oxidoreductase 1
Nrf2	nuclear factor erythroid 2-related factor 2
PBMC	peripheral blood mononuclear cell
PC	Protein carbonyls
PERK	protein kinase R-like ER kinase
PET	Positron emission tomography
PD	Parkinson disease
PKR	protein kinase R
PI3K	phosphoinositide 3-kinases
PQC	protein quality control
RCAN1	regulator of calcineurin
ROS	reactive oxygen species
Sirt1	NAD-dependent deacetylase sirtuin
SOD1	superoxide dismutase 1
ORF	upstream open reading frames
Ub	ubiquitin
UCH-L1	ubiquitin carboxy-terminal hydrolase L1
UPR	unfolded protein response
UPS	ubiquitin proteasome system
USP	ubiquitin specific peptidase
XBP1	X box-binding protein 1
